# Liquid Biopsy and Circulating Biomarkers for the Diagnosis of Precancerous and Cancerous Oral Lesions

**DOI:** 10.3390/ncrna8040060

**Published:** 2022-08-10

**Authors:** Giuseppe Gattuso, Salvatore Crimi, Alessandro Lavoro, Roberta Rizzo, Giorgia Musumarra, Simona Gallo, Flavia Facciponte, Sabrina Paratore, Angela Russo, Roberto Bordonaro, Gaetano Isola, Alberto Bianchi, Massimo Libra, Luca Falzone

**Affiliations:** 1 Department of Biomedical and Biotechnological Sciences, University of Catania, 95123 Catania, Italy; 2 Department of General Surgery and Medical Surgery Specialties, University of Catania, 95123 Catania, Italy; 3 Medical Oncology Unit, ARNAS Garibaldi, 95122 Catania, Italy; 4 Department of General Surgery and Surgical-Medical Specialties, School of Dentistry, University of Catania, Via S. Sofia 78, 95124 Catania, Italy; 5 Research Center for Prevention, Diagnosis and Treatment of Cancer, University of Catania, 95123 Catania, Italy; 6 Epidemiology and Biostatistics Unit, IRCCS Istituto Nazionale Tumori “Fondazione G. Pascale”, 80131 Naples, Italy

**Keywords:** liquid biopsy, oral cancer, circulating biomarkers, qPCR, ddPCR, NGS, ctDNA, epigenetics, miRNAs, Exosomes

## Abstract

Oral cancer is one of the most common malignancies worldwide, accounting for 2% of all cases annually and 1.8% of all cancer deaths. To date, tissue biopsy and histopathological analyses are the gold standard methods for the diagnosis of oral cancers. However, oral cancer is generally diagnosed at advanced stages with a consequent poor 5-year survival (~50%) due to limited screening programs and inefficient physical examination strategies. To address these limitations, liquid biopsy is recently emerging as a novel minimally invasive tool for the early identification of tumors as well as for the evaluation of tumor heterogeneity and prognosis of patients. Several studies have demonstrated that liquid biopsy in oral cancer could be useful for the detection of circulating biomarkers including circulating tumor DNA (ctDNA), microRNAs (miRNAs), proteins, and exosomes, thus improving diagnostic strategies and paving the way to personalized medicine. However, the application of liquid biopsy in oral cancer is still limited and further studies are needed to better clarify its clinical impact. The present manuscript aims to provide an updated overview of the potential use of liquid biopsy as an additional tool for the management of oral lesions by describing the available methodologies and the most promising biomarkers.

## 1. Introduction

According to the Globocan Cancer Observatory (International Agency for Research on Cancer—IARC, Lyon, France), the number of new cases of oral cancer in 2020 for both sexes and all ages was about 377,000 cases, and about 177,700 deaths were recorded in the same year, highlighting how oral cancer represents a growing public health problem. Oral cancer is most frequently diagnosed in Asia (65.8%), followed by Europe (17.3%), North America (7.3%), Latin America and the Caribbean (4.7%), and Africa (3.8%). Similarly, the mortality rate is higher in Asia (74%), followed by Europe (13.8%), Africa (4.6%), Latin America and the Caribbean (4.2%), and North America (2.8%) [1]. Both the incidence and mortality rates are higher in males than in females with a 2:1 ratio [2].

Of note, oral cancer affects different areas of the oral cavity including the lips, tongue, hard and soft palate as well as the buccal mucosa and gums [3]. It is defined as a subgroup of head and neck cancer (HNSC) and the most common form is oral squamous cell carcinoma (OSCC), which accounts for 90% of all oral cancer cases [4]. Importantly, OSCC often arises from pre-existing oral disorders such as leukoplakia and oral lichen planus, and it is generally characterized by a poor prognosis and a high mortality rate [5]. In addition, OSCC shows complex pathogenesis due to the involvement of multiple molecular mechanisms, gene mutations, and altered levels of proteins and metabolites [6].

An increasing number of risk factors have been associated with the development of oral cancer. As widely documented in the literature, there is a strong correlation between tobacco use and alcohol consumption and the incidence of oral cancer [7,8,9]. Additional risk factors include pre-cancerous oral lesions, *Human papillomavirus* (HPV) infection, pesticide exposure, genetic background, nutritional deficiencies caused by a poor diet and a weakened immune system, and chronic inflammation [10,11,12,13,14,15].

Despite the advancement of surgical and pharmacological treatments [16,17,18], oral cancer still represents one of the most impairing and deadly tumors, with an overall 5-year survival rate of ~50%. This poor survival rate is mainly due to the late diagnosis of oral cancer and the lack of effective diagnostic and prognostic biomarkers [19]. Therefore, there is an urgent need to identify novel effective proteins, genetic and epigenetic factors associated with oral cancer development, and patient prognosis [20].

At present, tissue biopsy and histopathological analyses are the gold standard methods for the diagnosis of oral cancer. However, these procedures have several limitations mainly related to their invasiveness and the need for sophisticated procedures performed by specialized professionals [21]. Tissue biopsies in the oral cavity are also not well tolerated by patients due to physical and functional distress. In addition, tissue biopsies are often not representative of the whole tumor bulk and histological examinations are time-consuming and expensive [22].

All of these limitations have prompted researchers to focus on the development of new tailored diagnostic and therapeutic approaches that could have a positive impact on patient survival. In this field, liquid biopsy is emerging as a novel tool potentially useful for the early detection and real-time monitoring of cancer patients in a minimally invasive manner [23,24]. Specifically, liquid biopsy could be used for the effective evaluation of circulating tumor cells (CTCs), circulating tumor DNA (ctDNA), and circulating microRNAs (miRNAs) as well as proteins and tumor-derived exosomes associated with the presence of tumor cells [25]. Although blood is the most commonly collected clinical specimen, other biological fluids can also be used including saliva, urine, cerebrospinal and synovial fluid, sputum, bronchoalveolar lavage fluid, exhaled breath condensate, amniotic fluid, seminal fluid, breast milk, nipple aspirate fluid, cervicovaginal fluid, pancreatic juice, tear fluid, and pleural effusions [26,27,28].

Aside from the non-invasive nature of liquid biopsy, other advantages compared to tissue biopsy are related to the possibility of providing a personalized snapshot of primary and metastatic tumors at different time points, performing a constant monitoring of tumor progression [29,30]. Second, liquid biopsy could be used to obtain relevant information for therapeutic decisions. Other advantages include the low cost, repeatability, reliability, and reproducibility of the analysis. In addition, it requires a shorter processing time than tissue biopsy [31].

Despite recent advances in this field, the impact of liquid biopsy on oral cancer diagnosis is still limited compared with other cancers and further studies should be undertaken to better clarify its clinical application [32]. As above-mentioned, the gold standard for the diagnosis of oral cancer is currently represented by tissue biopsy and histopathological analyses. Aside from these invasive methods, liquid biopsy could be used as an additional tool for the detection of tumor-related markers potentially being applied for the development of screening programs for the individual at risk for this tumor or for the monitoring of patient prognosis and response to treatments [29].

On these bases, the present review article aims to provide an update on the use of liquid biopsy as a diagnostic tool for oral cancer by describing the new available technologies and focusing on potential genetic, epigenetic, and protein biomarkers for the early diagnosis and better management of this malignancy.

## 2. Liquid Biopsy

**Figure 1:**
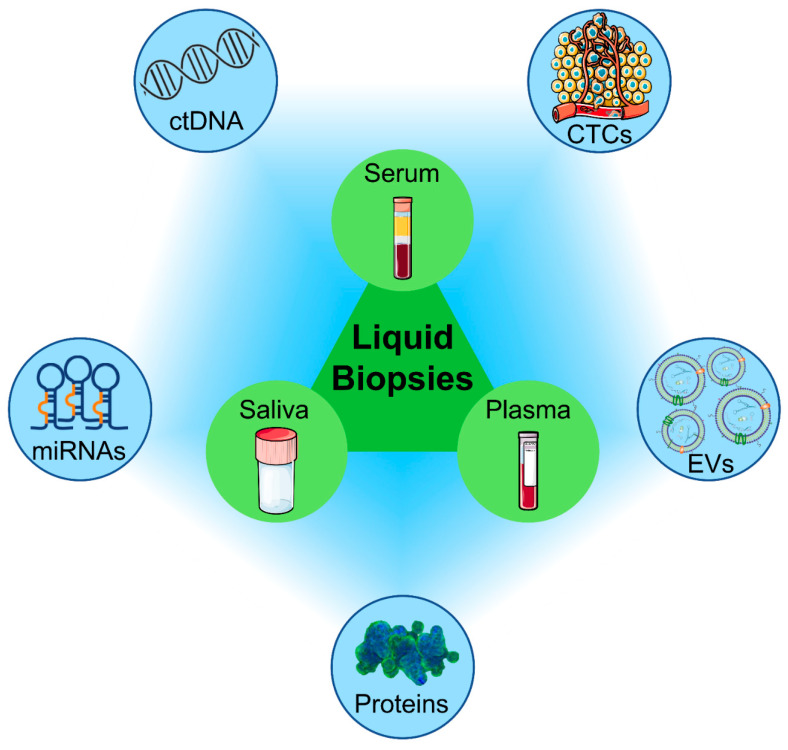
Liquid biopsy-based biomarkers. Abbreviations: CTCs—circulating tumor cells; ctDNA—circulating tumor DNA; miRNAs—microRNA; EVs—extracellular vesicles.

As already mentioned, liquid biopsy has recently attracted strong interest in the scientific community as an alternative diagnostic method to tissue biopsy. This non-invasive approach allows clinicians to display the tumor heterogeneity as well as the prognosis of cancer patients and the efficacy of anticancer treatments, thus improving the clinical assessment of various tumors including oral cancer [33,34]. In addition, it provides a better understanding of prognosis, resistance mechanisms, and disease recurrence, representing a valuable starting point for personalized medicine [35,36]. Although liquid biopsy was originally based on the detection of CTCs, in the last few years, it has been also extended to the analysis of ctDNA and miRNAs as well as proteins and extracellular vesicles such as exosomes (Figure 1) [37,38,39,40].

Of note, during the formation and growth of the primary tumor, cancer cells can migrate into the bloodstream and release several tumor-derived components that can be isolated from blood-based liquid biopsy and used as promising circulating biomarkers for the early detection of oral malignancies [41]. Peripheral blood is the major source of non-solid biological tissue used to obtain key information about tumorigenesis and metastasis. In addition, blood samples can be used to differentiate early- and advanced-stage cancer patients as well as to establish the patients’ response to treatment and disease progression [42]. Interestingly, although ctDNA can be detected in serum, the use of plasma is preferred to avoid contamination caused by leukocyte lysis and its adhesion to blood clots [43]. Peripheral blood samples (15–20 mL) can be obtained with a minimally invasive venipuncture. Generally, the specimen is collected into tubes containing anticoagulants such as ethylenediaminetetraacetic acid (EDTA), or tubes with specific reagents to prevent leukocyte lysis and ctDNA contamination [44,45]. After collection, the sample should be processed within six hours. Specifically, peripheral blood must be centrifuged at 2000× *g* (10 min) to separate plasma from whole blood. Then, the supernatant is centrifuged at 16,000× *g* (10 min) to remove cellular debris and the obtained plasma can be frozen into 2.0 mL tubes at −20 °C for up to three months. Alternatively, it is possible to store the samples at −80 °C for longer periods [46]. Aside from peripheral blood, other body fluids such as saliva can be used to detect circulating biomarkers [47].

Saliva is an acidic biological fluid (pH 6–7) secreted by the major salivary glands such as parotid, submandibular, and sublingual ones, and several minor glands including labial, lingual, buccal, and palatal [48]. Under physiological conditions, the daily production of saliva ranges from 500 to 1500 mL [49]. It is composed of 99% water, 0.3% proteins, 0.2% electrolytes (potassium, calcium, and magnesium), and organic substances (amylase, lysozyme, peroxidase, lipase, and mucins) [50]. It is well-known that saliva plays a critical role in several biological functions including lubrification, mastication, tasting, swallowing, digestion, perception of temperature, and touch. At the same time, it ensures the maintenance of oral cavity homeostasis, showing antibacterial, antifungal, and antiviral properties [51,52]. Similar to blood, saliva can be considered “*a mirror of the body*” as it reflects the physiological conditions and pathological changes occurring in the oral cavity and even in the entire body [53]. The collection of salivary samples, obtained after stimulation or not, is a fast, cost-effective, and non-invasive procedure because it does not require needles and specialized personnel. In fact, it can easily be obtained even from children and anxious subjects [54,55]. In addition, it is possible to collect a large volume of samples that are useful for different analyses and their repetition. Specifically, salivary samples should be collected in the morning after 12 h of fasting and immediately packed on ice or with protein stabilizing agents to protect the transcriptome and proteome [56,57]. Then, the sample must be centrifuged at 10,000–14,000× *g* (10–25 min) to remove the cellular debris and mucus. Finally, the obtained supernatant can be fractionated into cryotubes and frozen at −80 °C until further analysis or at 4 °C for processing within 3–6 h after collection [58].

Recently, an increasing number of studies have focused their attention on peripheral blood and salivary biomarkers as potential diagnostic tools for the early detection of oral cancer, highlighting the advantages of liquid biopsy compared to post-operative solid samples [54,58,59]. However, liquid biopsy-based biomarkers have not yet been validated in clinical practice due to some issues related to the sensitivity, specificity, and lack of standardized methods [60]. In this field, the future challenges will be to combine the analyses of peripheral blood and salivary biomarkers to ensure higher sensitivity and specificity.

## 3. Diagnostic Platforms for the Analysis of Liquid Biopsy Samples

**Figure 2:**
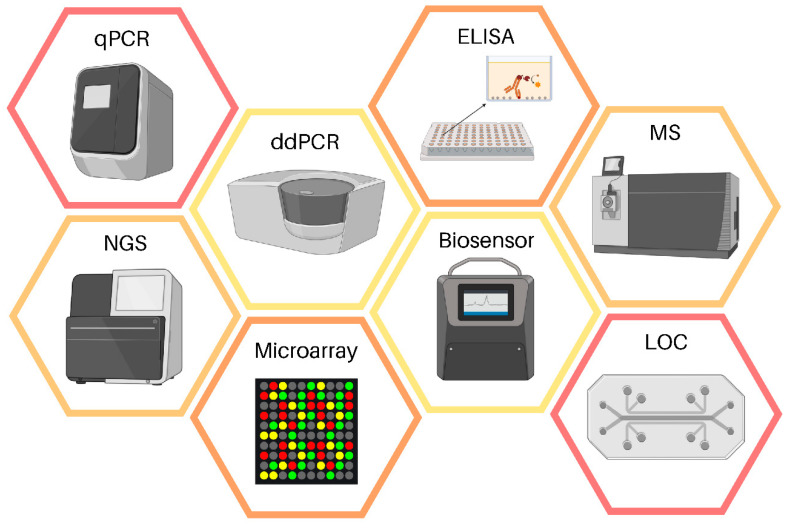
Platforms for the analysis of liquid biopsy samples. Abbreviations: qPCR—quantitative real-time polymerase chain reaction; ddPCR—droplet digital PCR; NGS: next generation sequencing; ELISA: enzyme-linked immunosorbent assay; LOC—lab-on-a-chip; MS—mass spectrometry.

Over the past few years, technological advances have provided new avenues for the early diagnosis of human diseases [61,62]. These advances have also improved the early diagnosis of cancer including that of oral cancer [63]. To date, several diagnostic techniques are available for the detection and discovery of circulating biomarkers (Figure 2). Among the most adopted technologies, real-time quantitative polymerase chain reaction (qPCR) is widely used in routine clinical practice and research settings [64]. Although qPCR represents the conventional method for the analysis of liquid biopsy samples, digital PCR (dPCR), droplet digital PCR (ddPCR), mass spectrometry (MS), and next generation sequencing (NGS) have recently emerged as more sensitive and specific techniques for the analysis of circulating biomarkers in cancer [59,65,66]. Aside from these technologies, other platforms with high diagnostic accuracy for the analysis of liquid biopsies have been proposed such as microarray, enzyme-linked immunosorbent assay (ELISA), biosensors, and lab-on-a-chip (LOC) [67,68,69].

### 3.1. qPCR

qPCR is the most widely used technique for the amplification and real-time quantification of nucleic acids. It can be used to quantify the expression of specific mRNAs and microRNAs (miRNAs) through their reverse transcription into complementary DNA (cDNA) and subsequent amplification in a procedure known as real-time reverse transcription quantitative PCR [70]. qPCR is based on the use of primers and fluorescent dyes or reporters such as SYBR green or TaqMan probes, respectively, which hybridize with the targets emitting a fluorescent signal [71]. Of note, the quantification of the expression level of genes can be relative or absolute. Specifically, relative quantification is based on the comparison between the concentration of the target gene and the concentration of the standard gene, while absolute quantification is performed using a calibration curve with known concentrations of the target [72].

Currently, qPCR represents the conventional method for the analysis of liquid biopsy samples and the discovery of novel oral cancer biomarkers. Using RT-qPCR, Maclellan SA et al. (2012) demonstrated that five miRNAs (miRNA-16, miRNA-let-7b, miRNA-338-3p, miRNA-223, and miRNA-29a) were differentially expressed in the serum of patients with pre-cancerous oral lesions compared to the healthy controls, however, the preliminary results obtained by Maclellan SA and colleagues need further validations on a wider cohort of samples [73]. Similarly, Oh SY and colleagues (2020) noted that the mRNA levels of six candidate genes including NGFI-A Binding Protein 2 (*NAB2*), cytochrome P450 family 27 subfamily A member 1 (*CYP27A1*), Nuclear Pore Complex Interacting Protein Family Member B4 (*NPIPB4*), Monoamine Oxidase B (*MAOB*), Sialic acid Acetyltransferase (*SIAE*), and Collagen Type III Alpha 1 (*COL3A1*), were significantly lower in salivary samples of OSCC patients compared to that of the control group [74]. Taken together, these findings highlight how qPCR can be used for the detection of circulating biomarkers potentially related to oral cancer. However, low-expressed biomarkers or a slight variation in their expression may not be correctly detected by qPCR [75].

### 3.2. ddPCR

ddPCR has recently emerged as one of the most powerful methodologies with high sensitivity and specificity, which can be used for the detection of cancer-associated biomarkers using liquid biopsy samples [76]. Compared to qPCR, this innovative tool has a higher sensitivity (0.01%) for absolute allele quantification, ctDNA somatic mutations, DNA methylation, and gene rearrangements [77]. Briefly, the ddPCR system is based on a water–oil emulsion of the reaction mix, in which the nucleic acid sample is fractionated into thousands of droplets (~20,000). Then, PCR amplification is performed within each portion and positive/negative fluorescent signals emitted by specific probes or dyes are detected from each droplet [61].

In recent years, several studies have proven the clinical application of ddPCR for the analysis of liquid biopsy samples and the discovery of circulating biomarkers related to oral cancer. Interestingly, van Ginkel JH and colleagues (2017) used ddPCR to detect ctDNA from the plasma samples of patients with head and neck squamous cell carcinoma (HNSCC). Specifically, the authors effectively detected the presence of a circulating *TP53* mutation in all of the tested samples, demonstrating the accuracy of ddPCR technology in detecting potential ctDNA blood-based biomarkers [78]. Recently, Crimi S et al. (2020) verified the diagnostic and prognostic role of miRNAs using plasma samples from oral cancer patients and healthy controls. Notably, the researchers observed that two miRNAs (hsa-miR-133a-3p and hsa-miR-375-3p) were significantly downregulated in cancer patients compared to the healthy controls, providing an innovative ddPCR-based protocol for the effective detection of new potential biomarkers from liquid biopsy samples [59].

### 3.3. NGS

NGS is a powerful technology based on sequencing by the synthesis of millions of DNA fragments in a short amount of time. In addition, this technique can also be used for RNA sequencing (RNA-Seq) to analyze small and non-coding RNAs, post-transcriptional modifications as well as changes in gene expression [79]. The most common platforms are Illumina and Ion Torrent, which use different specific sequencing approaches and signal detection methods [80]. NGS technology has provided a remarkable improvement in cancer diagnosis and management due to its high sensitivity in the detection of a wide board of known and unknown cancer-related mutations by analyzing the whole sequence of target genes (mutant allele fraction <1%) [81].

To date, NGS, along with ddPCR, is considered the most promising technique for the analysis of liquid biopsy samples and the detection of potential circulating biomarkers. In this field, Chang YA et al. (2018) applied RNA-Seq technology to plasma samples from oral leukoplakia/OSCC patients and healthy controls. Interestingly, the authors identified a group of three differentially expressed miRNAs (miR-222-3p, miR-150-5p, and miR-423-5p), which may represent potential biomarkers that are predictive for the malignant progression of oral lesions [82]. Subsequently, Cui Y and colleagues (2021) evaluated the efficacy of NGS for the surveillance of OSCC. Specifically, using plasma and saliva samples collected before and after surgery, the authors detected ctDNA in patients with recurrent cancer, highlighting the potential application of NGS technology for the prognosis of patients with oral cancer [83].

### 3.4. Microarray

Microarray represents a valuable biomedical platform with several applications, ranging from the evaluation of gene expression to DNA methylation and non-coding RNA expression profiles [84]. DNA microarrays are based on the principle of complementarity and in situ hybridization, which allows for the evaluation of thousands of genes in a single assay. Notably, the DNA fragments are collected on a solid surface where they can bind with probes, resulting in the emission of a fluorescence signal. Regarding RNA, reverse-transcription into cDNA is mandatory to detect and quantify the targets [85]. Generally, microarrays are used for the identification of single-nucleotide polymorphisms, gene mutations, and genes related to drug resistance in tissue biopsies [86].

Interestingly, microarrays have also been applied to liquid biopsy samples for the detection of oral cancer-related biomarkers. For example, Salazar C et al. (2014) analyzed salivary samples using microarray technology and identified three differentially expressed miRNAs (miR-9, miR-134, and miR-191) in HNSCC patients and healthy subjects [87]. Similarly, He L et al. (2020) found that the expression profile of miR-24-3p was altered in the saliva-derived exosomes from OSCC patients compared to the controls, indicating that microarray technology could be useful for the discovery of new circulating biomarkers [88].

### 3.5. ELISA

ELISA is an immunoenzymatic assay widely used in both research and clinical settings. It is based on antigen-antibody binding, which allows for the detection and quantification of antibodies, antigens, proteins, and other peptides such as glycoproteins and hormones [89]. Of note, it is possible to distinguish several types of detection methods including direct, indirect, sandwich, and competitive ELISA, which are characterized by different steps depending on the molecule of interest [90]. Due to their high throughput and sensitivity, ELISA assays represent the gold standard for the detection of various circulating tumor markers such as prostate-specific antigens (PSAs) and carcinoembryonic antigen (CEAs) [91].

Interestingly, ELISA may also be used to identify potential oral cancer biomarkers. Notably, Sivadasan P and colleagues (2020) focused their attention on the salivary proteomic profile of patients with dysplastic leukoplakia and OSCC. In particular, the ELISA assay showed that the protein levels of Cluster of Differentiation 44 (CD44), S100 Calcium Binding Protein A7 (S100A7), and S100 Calcium Binding Protein P (S100P) were significantly altered compared to the saliva samples from healthy subjects [92]. Similarly, Lotfi A and colleagues (2015) observed that the Matrix Metalloproteinase 2 (MMP2) and Matrix Metalloproteinase 9 (MMP9) expression levels were higher in the serum of OSCC patients than in the controls [93]. Overall, these results suggest that the ELISA assay is an effective tool for the detection of new oral cancer-associated biomarkers, which could improve the early diagnosis and management of this malignancy.

### 3.6. Biosensors

Biosensors are analytical tools used to detect several biological molecules including nucleic acids, enzymes as well as antibodies and antigens. Specifically, this device consists of a receptor able to bind the target and a transducer, which allows the conversion of the biochemical signal into an electrical signal [94]. Depending on the detection method, biosensors can be classified into six main types: electrochemical biosensors, surface plasmon resonance (SPR) biosensors, colorimetric biosensors, surface-enhanced Raman scattering (SERS) biosensors, immunofluorescence biosensors, and nuclear magnetic resonance (NMR) biosensors [95]. It is well-known that biosensors, thanks to their specificity and cost-effectiveness, are a valuable tool for the diagnosis of various diseases such as diabetes mellitus, cardiovascular diseases, and viral infections [61,96].

Of note, biosensors can also find application for the early diagnosis of oral cancers. As reported by Dong T and Pires NMM (2017), optical microfluidic biosensors show a high sensitivity for the detection of interleukin-8 (IL-8) in saliva samples, a pro-inflammatory cytokine involved in the onset of oral cancer [97]. Moreover, Tofighi FB and colleagues (2021) demonstrated that electrochemical biosensors were capable of detecting salivary levels of the cytokeratin-19 fragment (CYFRA 21.1), a tumor biomarker for HNSCC [98]. Taken together, these results suggest that biosensor technology could represent a promising tool for the diagnosis of oral cancer using liquid biopsies.

### 3.7. LOC

LOC technology integrates several analytical laboratory procedures on a single chip, providing a miniaturized and automated system for the detection of cellular and molecular elements. Among the various devices developed, the microfluidic-based system represents the most used, as characterized by the easy manipulation and suitability of cell separation [99]. LOC has recently attracted growing interest for its potential application in cancer diagnosis. In fact, this microfluidic engineering technology allows for the detection of CTCs, ctDNA, miRNAs, and proteins in a short time using small amounts of biological fluid samples. Moreover, these low-cost devices guarantee a high sensitivity and specificity in clinical settings [100].

In the last decade, LOC platforms have also been tested for the screening and diagnosis of oral cancer. For example, Gau V and Wong D (2007) developed the Oral Fluid Nano-Sensor Test (OFNASET) technology for the detection of liquid biopsy-based biomarkers. In particular, the authors have demonstrated that this microfluidic-based LOC system showed high sensitivity and specificity for several circulating biomarkers (miRNAs and proteins), paving the way for the development of an effective technology for the early diagnosis of oral malignancies [101].

### 3.8. Other Analytical Techniques

Besides the aforementioned methods, other analytical techniques could also represent a valuable alternative for the discovery of new circulating biomarkers in oral cancer. Among these, electric field-induced release and measurement (EFIRM) is an electrochemical sensing technology that can be used to analyze several body fluids. The most important advantages of this technique are represented by the capability to disrupt and release the content of extracellular vesicles (mRNA, miRNA, and proteins) and the short detection time (~10 min) of circulating biomarkers [102,103]. Another technique is 2-dimensional gel electrophoresis (2DGE), a top–down platform that allows for the visualization of complex protein mixtures. 2DGE could be applied for the analysis of liquid biopsy samples including saliva, urine, plasma, and serum [104]. Another useful technique for the evaluation of protein biomarkers is mass spectrometry (MS). Generally, MS is associated with SELDI-TOF (surface-enhanced laser desorption ionization-time of flight) and LC-MS (liquid chromatography-mass spectrometry), which play a key role in the desorption/ionization and measurement of proteins extracted from body fluids [105]. In addition, iTRAQ (isobaric tags for relative and absolute quantification reagents) could represent a valuable approach for the analysis of the plasma proteome [106]. However, despite recent technological advances, further studies are needed to develop standardized methods for the detection of liquid biopsy-based biomarkers.

## 4. Molecular Biomarkers

Although histological investigations on tissue biopsies still represent the gold standard for the diagnosis of oral cancer, the clinical relevance of circulating tumor biomarkers for the early detection of cancer as well as for the monitoring of treatments and patient prognosis was widely demonstrated in the last few years [107,108]. The detection of circulating biomarkers in liquid biopsy samples reflects the genetic and epigenetic alterations of both the tumor and its microenvironment, providing new information for the identification of novel biomarkers and therapeutic targets in the era of precision medicine. Specifically, the analysis of ctDNA levels, DNA methylation, and point mutations may improve the clinical assessment of cancer and the choice of therapeutic strategies [109]. At the same time, the detection of altered miRNA levels in body fluids such as blood and saliva could represent a promising tool for the diagnosis and prognosis of several malignancies including oral cancer [110].

### 4.1. ctDNA

Circulating free DNA (cfDNA) refers to the extracellular DNA released into the bloodstream by apoptotic and necrotic cells under both physiological and pathological conditions. Usually, cfDNA has a limited half-time (~15 min) because it is rapidly degraded by circulating endonucleases or phagocytosed by macrophages. In tumors, the rapid turnover of cancer cells causes a constant release and accumulation of cfDNA and ctDNA in the tumor microenvironment and in body fluids [111,112]. Of note, ctDNA can be a single- or double-stranded DNA fragments (130–140 bp), representing 0.1–10% of the total cfDNA. ctDNA is released by apoptotic and necrotic tumor cells, but also by living and circulating tumor cells [113,114]. Moreover, ctDNA is distinguished from cfDNA due to cancer-related modifications such as somatic mutations, alterations of the methylation status, and copy-number variations [115].

It is known that ctDNA can be found into the bloodstream and other body fluids including saliva, urine, and cerebrospinal fluid [116]. As widely described in the literature, the detection of ctDNA in liquid biopsy can reflect the genetic and epigenetic alterations in tumor tissue samples [117]. Moreover, many cancer characteristics (size, stage, location, vascularity, and treatment response) are correlated with the ctDNA concentrations [118,119]. Although the ctDNA levels are lower than cfDNA, several technologies have been developed to identify only the ctDNA. To date, qPCR and fluorescent assays represent the standard methods for the detection of ctDNA. On the other hand, ddPCR and NGS have recently been proposed as more sensitive and specific technologies for the analysis of ctDNA in liquid biopsy samples [120].

Over the years, body fluid-derived ctDNA has attracted strong interest as a diagnostic biomarker for several malignancies including lung, breast, pancreatic, colorectal, and ovarian cancers [121,122,123,124,125]. Furthermore, the analysis of ctDNA could be useful as a predictive factor in managing cancer treatment, post-treatment surveillance, and the development of personalized medicine [126].

In this field, many studies have also investigated ctDNA as a novel biomarker for oral cancer. Wang Y and colleagues (2015) conducted a study on oral cancer patients (with tumor affecting larynx, oropharynx, and hypopharynx) to measure the ctDNA levels in saliva and plasma samples. Specifically, ctDNA was detected in both early- and late-stage patients, showing higher specificity for saliva than plasma (100% and 80%, respectively). The same group also demonstrated that post-surgical detection of ctDNA was strictly related to disease recurrence, suggesting the utility of ctDNA for oral cancer follow-up and surveillance [127]. Similarly, Sukhija H et al. (2015) showed that the identification of circulating mutations in ctDNA obtained from liquid biopsy could represent an adjuvant diagnostic tool in OSCC. Notably, the researchers found that salivary samples from OSCC patients were positive for a C-deletion in exon 4 codon 63 of the *TP53* gene, while no mutation was observed in the healthy volunteers [128]. Interestingly, Perdomo S and collaborators (2017) focused on ctDNA mutations previously identified in tumor tissues. Using plasma and oral rinse samples, the authors detected ctDNA mutations affecting *TP53*, Notch Homolog 1 (*NOTCH1*), Cyclin-Dependent Kinase Inhibitor 2A (*CDKN2A*), Caspase 8 (*CASP8*), and Phosphatase and Tensin Homolog (*PTEN*) genes in 42% of HNC patients [129]. In another study by Mes SW et al. (2020), somatic mutations and copy number variations were detected in the plasma of HNSCC patients (67% and 52%, respectively), while HPV-DNA was detected in all cancer patients with HPV-positive tumors. Specifically, the authors demonstrated that the detection rate of ctDNA was increased (78%) by combining the analysis of somatic mutations, copy number variations, and HPV-DNA, indicating that multiparameter molecular analyses could improve the early diagnosis of oral malignancies [130].

Taken together, these findings highlight the potential application of ctDNA as a novel diagnostic biomarker for oral cancer detection. Although several methods have been proposed in the last few years, the major challenge will be to develop cost-effective and highly sensitive technologies to detect ctDNA levels at early cancer stages and simultaneously analyze different mutations.

### 4.2. miRNAs

miRNAs are a large group of small endogenous single-stranded RNAs (18–24 nucleotides in length) that control the expression of target genes at the post-transcriptional level [131]. Binding to 3′-untranslated regions (3′-UTR) of the target mRNA, these non-coding RNAs could promote mRNA degradation or inhibit mRNA translation into proteins [132]. Interestingly, a single miRNA can target several mRNAs and a specific mRNA can be targeted by different miRNAs depending on the complementary existing between the miRNA “*seed region*” and the 3′-UTR of the targeted mRNA [133].

miRNAs play crucial roles in several biological processes including cell cycle regulation, differentiation, apoptosis, immune response, and homeostasis. However, the aberrant expression of these small ncRNA molecules has been associated with various pathological conditions including cancer development and progression [134,135]. Of note, miRNAs can regulate the expression of target genes involved in cancer biology by acting as oncogenes or tumor suppressors [136]. Over the years, miRNAs have been widely associated with cancer cell proliferation, invasion, metastasis, and angiogenesis, suggesting that they may serve as therapeutic targets for novel effective anti-cancer treatments [137,138]. Since miRNAs are highly stable in different biological fluids (serum, plasma, and saliva), they have also emerged as potential circulating biomarkers [139].

The expression levels of miRNAs in the peripheral blood and saliva samples obtained from oral cancer patients have been investigated, highlighting that miRNAs could be used for both diagnostic and prognostic purposes [59,140,141]. In this field, Mazumder S et al. (2019) reviewed the most recent studies on circulating miRNAs as liquid biopsy-based biomarkers and reported that miRNA-134, miRNA-146a, and miRNA-338 were strictly related to oral cancer progression, while miRNA-7d, miRNA-21, miRNA-150, and miRNA-371 showed potential prognostic value [142]. In addition, as described by Patil S and Warnakulasuriya S (2020), several miRNAs including miRNA-9, miRNA-29c, miRNA-223, and miRNA-187 were downregulated in the blood of HNC patients, while miRNA-let-7c, miRNA-17, miRNA-20a, miRNA-22, miRNA-29a, miRNA-24-3p, miRNA-29b, miRNA-103, miRNA-191-5p, miRNA-196a, miRNA-200b-3p, miRNA-374b-5p, miRNA-375, miRNA-425-5p, miRNA-483-5p, miRNA-572, miRNA-638, and miRNA-1234 were upregulated [143]. Another systematic review on the expression levels of salivary miRNAs conducted by Al Rawi N et al. (2021) revealed that several miRNAs were overexpressed (miRNA-21, miRNA-31, miRNA-122, miRNA-134, miRNA-184, miRNA-191, miRNA-196a, miRNA-196b, miRNA-412, miRNA-512, and miRNA-8392) or downregulated (miRNA-let-7a, miRNA-27, miRNA-34, miRNA-92, miRNA-124, miRNA-125a, miRNA-136, miRNA-139, miRNA-145, miRNA-200a, and miRNA-205) in OSCC patients compared to healthy subjects [144].

In the last year, a growing number of studies have further investigated the potential clinical application of miRNAs as circulating biomarkers for the early diagnosis of oral cancer. Baber S et al. (2021) focused on miRNA-153 and miRNA-455-5p, which have been described to play a critical role in oral cancer development. Interestingly, the authors observed that the expression levels of miRNA-153 were decreased in the blood samples of OSCC patients than in healthy individuals (−1.97-fold), while the expression levels of miRNA-455-5p were increased (2.5-fold), suggesting that the detection of these liquid biopsy-based miRNAs could represent an adjuvant diagnostic tool [145]. Similarly, Nakamura K and colleagues (2021) evaluated the expression levels of cancer-associated miRNAs in the serum samples from OSCC patients. Notably, the microarray and qRT-PCR assays showed that miR-5100 expression was significantly reduced in the OSCC group compared to the controls, while miRNA-19a and miRNA-20a were increased [146]. Another case-control study was conducted on a group of OSCC patients and healthy volunteers by Bolandparva F and colleagues (2021). The authors found that miRNA-138 and miRNA-424-5p were differentially expressed between the two groups. Specifically, the blood levels of miRNA-138 were lower in the OSCC patients than in the healthy controls (−3.05-fold), while miRNA-424-5p was upregulated in the disease group (1.96-fold) [147].

Aside from peripheral blood, saliva is another promising liquid biopsy sample for the detection of new potential biomarkers. In this regard, Mehterov N and colleagues (2021) evaluated the expression levels of miRNAs in the saliva supernatant of OSCC patients. Notably, qRT-PCR showed that miRNA-30c-5p was significantly downregulated in the OSCC group compared to the controls with a sensitivity of 86% and specificity of 74% [148]. Moreover, Romani C and colleagues (2021) performed a genome-wide analysis of salivary miRNAs in a cohort of OSCC patients and healthy subjects, identifying a panel of three miRNAs (miRNA-106b-5p, miRNA-423-5p, and miRNA-193b-3p) with higher expression levels in the OSCC group than in the controls. At the same time, miRNA-423-5p was inversely correlated to disease-free survival (DFS) and its salivary levels were significantly reduced after surgery, indicating that this miRNA could represent a promising circulating biomarker for oral cancer diagnosis and follow-up [149]. Of note, Cheng AJ et al. (2021) analyzed a panel of cancer-associated miRNAs in the saliva samples of HNSCC patients, oral precancerous lesion patients, and healthy individuals. The differentially expressed miRNAs were also investigated in cancer and normal tissues by using two independent cohorts, The Cancer Genome Atlas (TCGA) and Gene Expression Omnibus (GEO) datasets. Specifically, the integrated analysis showed that miRNA-196b was upregulated both in the saliva of HNSCC/oral precancerous lesion patients and tumoral tissues [150]. Similarly, Falzone L and collaborators (2019) performed a broad computational analysis of all existing miRNA expression datasets for oral cancer patients, highlighting a panel of 11 upregulated or downregulated miRNAs for the early diagnosis of oral cancer and the prediction of patient prognosis [151].

Table 1 summarizes the liquid biopsy-based miRNAs as potential circulating biomarkers of oral cancer. Overall, the use of liquid biopsy to detect deregulated miRNAs may represent a promising avenue for the early diagnosis of oral cancer. However, further multicenter clinical trials with a large sample size should be performed to validate its application in routine clinical practice.

## 5. Protein-Based Biomarkers

Protein biomarkers have significantly improved the management of different tumors, ameliorating both the diagnostic and follow-up strategies. Despite the identification of several protein biomarkers such as carbohydrate antigen 19-9 (CA19.9), cancer antigen 125 (CA125), carcinoembryonic antigen (CEA), etc., no effective biomarkers for the early diagnosis of oral cancer have been validated yet. Over the years, a growing number of studies have focused on the proteomic analysis of several body fluids (peripheral blood, serum, plasma, saliva, sputum, and urine) to provide a better understanding of the molecular mechanisms associated with oral cancer and discover new potential protein biomarkers [152,153]. In this field, inflammation-related proteins including cytokines and C-reactive protein (CRP) and proteases such as matrix metalloproteinases (MMPs) have recently been proposed as potential salivary biomarkers for oral cancer [154]. Other studies have focused the attention on Cluster of Differentiation 44 (CD44) and Cancer Antigen 125 (CA-125), cytoskeleton fragments including CYFRA-21-1 and Tissue Polyoeotide-Specific Antigen (TPS) as well as intracellular proteins such as Mac-2 Binding Protein (M2BP), demonstrating how these proteins could represent interesting biomarkers for the early detection of oral malignancies [155]. A careful review of the studies on protein biomarkers for the diagnosis and management of oral cancer is provided in the following subsections.

### 5.1. Cytokines

Cytokines are small soluble glycoproteins (<30 kDa molecular weight) with a short half-life usually released in response to a stimulus such as infection or inflammation. Cytokines are important regulators of the immune response, controlling the differentiation, proliferation, migration, and apoptosis of target cells [156]. The relationship between chronic inflammation and cancer has been widely documented over the years (157). Pro-inflammatory cytokines such as interleukin 1 (IL-1), interleukin 6 (IL-6), interleukin 8 (IL-8), interleukin 10 (IL-10) as well as transforming growth factor β (TGF-β), and tumor necrosis factor α (TNF-α) are produced by the cells of the tumor microenvironment, playing a key role in cancer initiation, growth, progression, and metastasis [157,158,159,160]. Conversely, interleukin 2 (IL-2) and interferon α (IFN-α) show anti-tumor properties including anti-proliferative and pro-apoptotic activities [161].

Cytokines have recently been investigated as new circulating biomarkers for the early diagnosis of oral cancer. Panneer Selvam N et al. (2015) observed that salivary IL-6 levels were significantly higher in OSCC and oral leukoplakia patients compared to the controls (132.88 ± 59.09 pg/mL, 52.14 ± 43.00 pg/mL, and 12.84 ± 9.68 pg/mL, respectively) [162]. Similarly, Aziz S et al. (2015) noted that the levels of IL-10 and interleukin 13 (IL-13) were enhanced in the saliva samples of OSCC patients [163]. Subsequently, Singh P and colleagues (2020) evaluated the clinical utility of other pro-inflammatory cytokines. The authors found that the concentrations of IL-8 and IL-1β were significantly increased in the saliva samples from OSCC patients than the healthy controls [164]. A recent study also highlighted that the salivary levels of interleukin 17A (IL-17), interleukin 17B (IL-17B), and TNF-α were strictly related to oral cancer progression [165]. These results suggest that the detection of salivary cytokine levels represents a valuable strategy for the development of novel non-invasive diagnostic tools for the identification of oral pre-cancerous lesions and to predict the development of advanced form oral squamous cell carcinoma.

### 5.2. CRP

CRP is a plasma protein encoded by the *CRP* gene, which is located on the short arm of chromosome 1. This conserved protein is secreted by hepatocytes, and it is composed of a cyclic structure of five identical subunits (~30 kDa) [166]. CRP is a protein involved in innate immunity and host defense against pathogens. Interestingly, serum CRP levels can increase rapidly (1000-fold within 48 h) in response to tissue damage or infection [167]. Over the years, the detection of circulating CRP level has attracted strong interest as a useful tool in clinical practice. Notably, it has been proposed as a diagnostic and prognostic biomarker for cardiovascular diseases and malignancies [168,169].

In this field, several studies have demonstrated the potential utility of CRP for the diagnosis of oral diseases. Metgud R and Bajaj S (2016) reported that salivary and serum concentrations of CRP were higher in OSCC and oral premalignant lesion patients compared to healthy individuals, highlighting how the increase in the CRP levels was strictly related to disease progression [170]. A similar result was obtained by Vankadara S et al. (2018), who observed higher serum levels of CRP in the OSCC patients (from 3.3 to 96 mg/L) than the oral premalignant lesion patients (from 0.8 to 53.9 mg/L) and the controls (from 0.1 to 18.3 mg/L) [171]. In line with these results, Knittelfelder O and co-workers (2020) recently showed that the CRP levels were inversely correlated with overall survival and cancer-specific survival, suggesting the clinical utility of this circulating protein as a prognostic marker for oral cancer [172].

### 5.3. Matrix Metalloproteinases

Matrix metalloproteinases (MMPs) are a family of 24 zinc-dependent endopeptidases involved in the degradation and remodeling of several components of the extracellular matrix [173]. MMPs are characterized by a pro-peptide sequence (~80 amino acids), a catalytic domain (~170 amino acids), a peptide linker, and a hemopexin domain (~200 amino acids) [174]. These proteolytic enzymes are secreted by different cell types (fibroblasts, osteoblasts, macrophages, neutrophils, and lymphocytes) and can be classified into several groups according to their substrates and structural domains [175]. MMPs are involved in different biological processes including angiogenesis, embryogenesis, morphogenesis, and tissue repair. However, the aberrant expression of MMPs seems to be strictly related to cardiovascular diseases as well as solid tumor progression and aggressiveness [176].

High levels of MMPs have been associated with poor overall survival in cancer patients. Therefore, a growing number of studies have focused their attention on the detection of these enzymes in liquid biopsies to investigate their clinical application as diagnostic and prognostic biomarkers for oral cancer. Hsin CH et al. (2014) performed a study on OSCC patients, analyzing the MMP levels. Using an ELISA assay, the authors revealed a positive correlation between MMP11 and disease progression [177]. Peisker A and colleagues (2017) performed a case-control study on the MMP9 protein levels, demonstrating that the salivary concentration of MMP9 was higher (+19.2%) in the OSCC patients compared to the healthy controls (healthy subjects) [178]. Similarly, another study showed that the OSCC patients had increased salivary levels of MMP1 compared to patients with premalignant disorders and to the control group, suggesting that this proteolytic enzyme was strictly associated with cancer progression [179]. Recently, Saleem Z et al. (2021) also highlighted that MMP12 was differentially expressed among the OSCC patients (mean value 14.92 ng/mL), oral mucous fibrosis patients (mean value12.53 ng/mL), and controls (mean value 0.82 ng/mL) with a specificity and sensitivity of 100% [180].

### 5.4. CD44

CD44 is a complex transmembrane adhesion glycoprotein (85–95 kDa) encoded by the *CD44* gene, which is located on chromosome 11 [181]. During the transcription process, exons 1–5 and 16–20 are spliced together, producing the standard isoform CD44s, while exons 6–15 are alternatively spliced to form the variant CD44 isoforms (CD44v). Of note, CD44s is characterized by three regions (extracellular, transmembrane, and cytoplasmatic domains), while the CD44v isoforms are composed of an additional membrane proximal domain [182]. As reported in the literature, CD44 plays a key role in many physiological processes such as cell–cell and cell–matrix adhesion, organ development, and hematopoiesis. The aberrant expression of this cell-surface glycoprotein seems to be involved in the growth and development of several tumor types [183].

In oral cancer, the detection of circulating CD44 levels has recently been proposed as a non-invasive prognostic method. In this field, Sawant S et al. (2018) showed that the serum CD44 levels were significantly increased in oral cancer patients than in the controls (251 ± 69.3 ng/mL and 178 ± 29 ng/mL, respectively). In addition, the aberrant expression of CD44 was inversely correlated to the overall survival of cancer patients [184]. Similarly, Shah K et al. (2018) observed that the expression of CD44v6 and CD44v10 was higher in the salivary samples from the OSCC patients compared to the healthy subjects, highlighting the potential utility of CD44v as a protein biomarker for the early detection of cancer [185].

### 5.5. CA-125

CA-125, also known as mucin 16 (MUC16), is a tumor-associated mucin glycoprotein with a high molecular weight, which is characterized by a tandem repeat region (~60 repeats) between the N-terminal and C-terminal domains [186]. Under physiological conditions, this cell-surface glycoprotein is expressed by the epithelial cells of several organs such as bronchial, tracheal, ocular, endometrial, and ovarian epithelial cells. However, CA-125 overexpression is also associated with the inhibition of natural killer cells and immune response, cancer cell proliferation, metastatic invasion, and poor prognosis [187].

Although CA-125 is mainly considered as a serum biomarker for the diagnosis of ovarian cancer, some studies have suggested that the detection of salivary CA-125 levels could be indicative for oral cancer lesions. For example, Nagler R and colleagues (2006) performed a case-control study to evaluate the salivary concentration of CA-125. Interestingly, the researchers observed a significant increase (400%) in the CA-125 levels in the OSCC group compared to the control group [188]. Similarly, Balan JJ and collaborators (2012) found that the mean salivary concentration of CA-125 in OSCC patients and the controls was 320.25 U/mL and 33.14 U/mL, respectively [189]. Taken together, these data support the potential role of salivary CA-125 evaluation in predicting the presence of oral cancer.

### 5.6. CYFRA 21-1

CYFRA 21-1 is a soluble proteolytic fragment of cytokeratin 19 (CK-19), a protein of 40 kDa that is expressed by epithelial cells. During cell apoptosis, CK-19 fragments are released into circulation due to caspase-3 activation. These soluble fragments can also be released during the proliferation of malignant cells [190]. Over the years, the detection of CYFRA 21-1 levels in different body fluids has been described as a promising diagnostic tool for several solid tumors, especially for lung, bladder, and stomach cancer [191,192,193].

Interestingly, CYFRA 21-1 could represent an interesting protein biomarker for the diagnosis of oral cancer. In particular, the serum CYFRA 21-1 levels showed a positive correlation with tumor depth, skin and bone invasion as well as the risk of metastasis in oral cancer patients [194]. Malhotra R and colleagues (2016) observed that serum and salivary levels of CYFRA 21-1 were significantly increased in the OSCC patients than in the controls (2.75-fold). The results highlight the high sensitivity (91%) of electro-chemiluminescent immunoassay (ECLIA) in the detection of CYFRA 21-1 as a cancer biomarker [195].

### 5.7. Tissue Polypeptide-Specific Antigen

Tissue polypeptide-specific antigen (TPS) is a soluble fragment of cytokeratin 18 (CK-18), an intermediate filament protein of the cytoskeleton. It is released during both apoptosis and neoplastic transformation processes [196]. Of note, TPS is a well-documented marker for the diagnosis and prognosis of several epithelial malignancies. In fact, it has been demonstrated that high TPS serum levels are associated with lung, ovarian, breast, and colorectal cancers [197,198,199,200].

The increase in the TPS levels in the saliva and serum samples seems to be associated with oral malignancies. Geng XF and colleagues (2013) evaluated the salivary levels of TPS in the OSCC patients and healthy controls, detecting a significant difference between the two groups (272.28 U/mL and 86.6 U/mL, respectively). These data demonstrate that the detection of TPS has high sensitivity (82.1%) and specificity (74.0%) for the diagnosis of oral cancer [201]. Subsequently, Barak V et al. (2015) investigated the serum levels of different tumor markers in HNC patients including TPS. Specifically, the authors observed that the TPS levels were increased before therapy, while a significant decrement in the TPS levels was observed after surgery or chemotherapy, suggesting that the lowering of TPS levels could be used as a prognostic biomarker for therapeutic efficacy in HNC patients [202].

### 5.8. M2BP

M2BP, also known as Lectin Galactoside-binding Soluble 3 Binding Protein (LGALS3BP), is a glycosylated protein of ~90 kDa. M2BP is characterized by seven N-glycosylation sites and it is mainly involved in cell adhesion and host defense [203]. Since M2BP can induce cytokine production in inflammatory processes, this glycoprotein and its glycan isomer have been proposed as serum biomarkers for the clinical evaluation of several pathologies [204,205,206]. Notably, M2PB expression levels are increased in lung and breast cancer [207,208].

Some studies have also evaluated the serum and salivary concentrations of M2BP in oral cancer patients. In particular, Weng LP et al. (2008) observed that the serum M2BP levels were significantly higher in the OSCC group than in the controls (8.06 ± 5.76 and 5.54 ± 5.1 µg/mL, respectively) [209] while Brinkmann O and colleagues (2011) found that the M2BP levels were increased in the saliva samples from OSCC patients, suggesting that M2BP could be an effective circulating biomarker for the early diagnosis of oral cancer [210].

### 5.9. Other Protein Biomarkers

Recently, proteomic analyses have been applied to discover novel reliable biomarkers for the early diagnosis of tumors [66]. Such analyses performed on liquid biopsy samples are effective in identifying new potential protein biomarkers for oral malignancies. Specifically, proteomics analyses have revealed that Zinc Finger Protein 510 (ZNF-510), Fibrinogen β chain (FGB), S100 calcium binding protein (S100), Transferrin (TF), Immunoglobulin Heavy Chain Constant Region γ (IGHG), Cofilin 1 (CFL1), S100 Calcium-Binding Protein A9 (S100A9), MAC-inhibitory protein (MAC-IP), Profilin (PFN), Catalase (CAT), Resistin (RETN), Gelsolin (GSN), Fibronectin (FBN), Angiotensinogen (AGT), Haptoglobin (HP), Complement Factor H (CFH), Fibrinogen α chain (FGA), α -1-antitrypsin (SERPINA1), and Heat Shock Protein α (HSP90α) showed higher expression levels in the saliva and serum samples of oral cancer patients compared to the controls [211,212,213,214,215,216,217]. Although these circulating proteins represent promising biomarkers for the diagnosis of oral cancer, further studies are needed to validate their sensitivity and specificity rates.

## 6. Exosome-Derived Biomarkers

Exosomes are small extracellular vesicles of endocytic origin with a diameter of 30–150 nm. These nanovesicles can be released by almost all cell types including epithelial cells, adipocytes, and fibroblasts. In addition, exosomes can be found in several body fluids such as saliva, blood, tears, amniotic fluid, breast milk, urine, and cerebrospinal fluid [218,219]. Of note, ultracentrifugation currently represents the standard method for the isolation of exosomes from cell culture supernatants or biological fluids [220].

The biogenesis of the exosome is a multi-step process that begins with the retraction of the endosomal membrane. Briefly, early endosomes are enriched with intraluminal vesicles (ILVs) which are transformed into late endosomes, also known as multivesicular bodies (MVBs). Then, MVBs fuse with the cell membrane to release ILVs or migrate to lysosomes and autophagosomes for degradation. When the ILVs reach the extracellular space, they take the name of exosomes [221]. Interestingly, several proteins and molecules are involved in the formation and secretion of exosomes including the Endosomal Sorting Complex Required for Transport (ESCRT-0, -I, -II, -III) and the Rab family of GTPases (Rab11, Rab27a, Rab27b, and Rab35). Moreover, tetraspanins and heat shock proteins (HSPs) have been described as part of the ESCRT-independent mechanism for the content assembly and release of exosomes [222,223].

The molecular content of exosomes is strictly related to the parental cells from which they originated and includes a variety of proteins, lipids, DNA fragments, long non-coding RNAs (lncRNAs), mRNAs, and miRNAs, which are transported to proximal cells or distant sites via the bloodstream [224]. Under physiological conditions, exosomes act as mediators of intercellular communication, signal transduction, and cell homeostasis. However, these nanovesicles have been also associated with tumorigenesis. Indeed, cancer-derived exosomes are released by cancer cells and promote cancer development, progression, invasion, and metastasis by interacting with the cells of the tumor microenvironment [225,226]. Since exosomes can be isolated from several body fluids, they have attracted growing interest as potential diagnostic and prognostic biomarkers in cancer [227,228,229].

In this field, recent studies have focused attention on the detection of exosome-derived miRNAs in liquid biopsy samples from oral cancer patients (Table 2). Gai C and colleagues (2018) used saliva samples from OSCC patients and healthy controls to evaluate the differential expression of extracellular vesicle-derived miRNAs. Specifically, the authors observed that the expression levels of miR-512-3p and miR-412-3p were higher in the cases compared to the controls. In addition, miR-302b-3p and miR-517-3p were found exclusively in OSCC patients [230]. Extracellular vesicle-derived miRNAs have been also investigated in HNSCC patients. Specifically, miR-491-5p, miR-630, and miR-1910-5p were significantly overexpressed in the plasma samples of cancer patients compared with those of the control group, while miR-27b-3p was downregulated. Interestingly, the expression levels of miR-491-5p were associated with the HNSCC patients’ overall survival and disease-free survival [231]. Recently, He T et al. (2021) showed that the evaluation of exosome-derived miR-130a expression levels could improve oral cancer diagnosis and prognosis. The researchers found that the miR-130a expression levels were higher in the plasma and tissue samples from the OSCC patients than in the controls. In addition, the expression of miR-130a was inversely correlated with the patients’ overall survival and recurrence-free survival [232].

Aside from miRNAs, proteomic analyses on extracellular vesicles obtained from liquid biopsy samples could represent an additional diagnostic tool for oral malignancies (Table 2). In this regard, Zlotogorski-Hurvitz A and colleagues (2016) conducted a case-control study on oral cancer patients and healthy controls using saliva samples for the isolation of exosomes and the evaluation of protein expression. Specifically, the authors observed that the expression of Cluster of Differentiation 63 (CD63) was increased in oral cancer patients compared to the controls. Conversely, the protein expression of Cluster of Differentiation 9 (CD9) and Cluster of Differentiation 81 (CD81) in the exosomes was downregulated in cancer patients [233]. Another study evaluated the expression levels of exosome-derived Lysyl Oxidase Like 2 (LOXL2) protein, demonstrating that LOXL2 was significantly overexpressed in serum samples of the HNSCC patients compared to healthy individuals [234]. In addition, Nakamichi E and colleagues (2021) investigated the exosomal expression levels of ALG-2-interacting protein X (Alix), a protein that has been detected in tumor tissues. Compared to the controls, the researchers found that Alix was overexpressed in both the saliva and serum samples from the OSCC patients, suggesting that this exosome-derived protein could be used as a potential biomarker for the early diagnosis of OSCC [235]. Interestingly, Guo H et al. (2021) recently performed the proteomic analysis of serum exosomes to measure the protein content and identify cancer-associated biomarkers. Notably, the researchers observed that CRP, von Willebrand factor (VWF), and Leucine-Rich α-2-Glycoprotein (LRG) were significantly increased in OSCC patients compared to the controls [236].

Overall, the results here presented demonstrate that the evaluation of the molecular cargo of liquid biopsy-derived exosomes could be useful to identify novel effective biomarkers for oral cancer. However, future studies will be necessary to further investigate the clinical relevance of exosome-derived miRNAs and proteins for the diagnosis and prognosis of this tumor.

## 7. Conclusions

Liquid biopsy has demonstrated a great potential as a non-invasive, rapid, and repeatable approach for the diagnosis and surveillance of oral cancer. According to the studies described in the present review article, the detection and analysis of biomarkers from peripheral blood and saliva (ctDNA, miRNAs, proteins, and exosomes) could significantly improve the current screening programs and diagnostic strategies, improving the early diagnosis and real-time monitoring of disease in the era of precision and personalized medicine. Although several technologies have been proposed over the years, the application of liquid biopsy in routine clinical practice is still limited due to some issues related to the sensitivity, specificity, and lack of standardized protocols. Overall, a future challenge will be to develop cost-effective and highly sensitive technologies for the detection of circulating biomarkers that are predictive for precancerous lesions or early-stage tumors. In addition, further clinical studies should be performed on a larger cohort of patients and controls in order to confirm the diagnostic and prognostic accuracy of circulating biomarkers and liquid biopsy in oral cancer.

## Figures and Tables

**Table 1:** The circulating miRNAs for the early diagnosis and surveillance of oral cancer.

Sample	Methodology	Biomarker	Ref.
Serum	qRT-PCR	miRNA-16, miRNA-let-7b (↑) miRNA-338-3p, miRNA-29a, miRNA-223 (↓)	[73]
Plasma	ddPCR	hsa-miRNA-133a-3p, hsa-miRNA-375-3p (↓)	[59]
Plasma	RNA-Seq, qRT-PCR	miRNA-150-5p, miRNA-423-5p (↑) miRNA-222-3p (↓)	[82]
Saliva	Microarray, qRT-PCR	miRNA-9 (↑) miRNA-134, miRNA-191 (↓)	[87]
Blood	qRT-PCR	miRNA-455-5p (↑) miRNA-153 (↓)	[145]
Serum	Microarray, qRT-PCR	miRNA-19a, miRNA-20a (↑) miRNa-5100 (↓)	[146]
Blood	qRT-PCR	miRNA-424-5p (↑) miRNA-138 (↓)	[147]
Saliva	qRT-PCR	miRNA-30c-5p (↓)	[148]
Saliva	Microarray, qRT-PCR	miRNA-106b-5p, miRNA-423-5p, miRNA-193b-3p (↑)	[149]
Saliva	qRT-PCR	miRNA-196b (↑)	[150]

Abbreviations: ddPCR—digital droplet PCR; qRT-PCR—quantitative real-time reverse transcription PCR; RNA-Seq—RNA sequencing; ↑—upregulated; ↓—downregulated.

**Table 2:** The exosome-derived biomarkers for oral cancer diagnosis.

Sample	Methodology	Exosome-Derived Biomarker	Ref.
Saliva	Microarray, qRT-PCR	miRNA-24-3p (↑)	[88]
Saliva	qRT-PCR	miRNA-512-3p, miRNA-412-3p, miRNA-302b-3p, miRNA-517-3p (↑)	[230]
Plasma	qRT-PCR	miRNA-491-5p, miRNA-630, miRNA-1910-5p (↑) miRNA-27b-3p (↓)	[231]
Plasma	qRT-PCR	miRNA-130a (↑)	[232]
Oral fluids	AFM, ELISA, WB	CD63 (↑) CD9, CD81 (↓)	[233]
Serum	WB	LOXL2 (↑)	[234]
Serum, Saliva	ELISA	Alix (↑)	[235]
Serum	ELISA, IHC, qPCR	CRP, VWF, LRG (↑)	[236]

Abbreviations: AFM—atomic force microscopy; Alix—ALG-2-interacting protein X; CD9—Cluster of Differentiation 9; CD6—Cluster of Differentiation 63; CD81—Cluster of Differentiation 81; CRP—C-reactive protein; ELISA—enzyme-linked immunosorbent assay; IHC—immunohistochemistry; LRG—leucine-rich α-2-glycoprotein; LOXL2—lysyl oxidase like 2; qPCR—quantitative real-time polymerase chain reaction; qRT-PCR—quantitative real-time reverse transcription PCR; VWF—von Willebrand factor; WB—Western blot; ↑—upregulated; ↓—downregulated.

## Data Availability

The data reported in the manuscript are available from the corresponding author on request. The original contributions presented in the study are publicly available. These data can be found at: www.pubmed.com (accessed on 1 April 2022).
